# Association between triglyceride glucose-body mass index and long-term adverse outcomes in individuals with heart failure: a retrospective cohort study

**DOI:** 10.3389/fnut.2025.1688566

**Published:** 2025-11-24

**Authors:** Yanan Shi, Chuanyu Gao, Yu Xu, Fang Yuan

**Affiliations:** Department of Cardiology, Fuwai Central China Cardiovascular Hospital, Zhengzhou, Henan, China

**Keywords:** heart failure, insulin resistance, poor prognosis, triglyceride glucose-body mass index, cardiology

## Abstract

**Background:**

Insulin resistance (IR) plays a pivotal role in the pathogenesis and progression of heart failure (HF), and may be exacerbated in advanced stages of HF, thereby perpetuating a deleterious cycle. The triglyceride glucose-body mass index (TyG-BMI), an established surrogate marker for IR, has been associated with adverse cardiovascular events. However, its prognostic significance in individuals diagnosed with HF has not been fully elucidated. The specific aim is to evaluate the association between the TyG-BMI index and long-term all-cause mortality and HF-rehospitalization in patients with heart failure, and to examine whether right ventricular dysfunction (RVD) modifies this association.

**Methods:**

This retrospective cohort study included 1,644 participants hospitalized with HF at Fuwai Central China Cardiovascular Hospital, of whom 850 (51.7%) had HF with preserved ejection fraction (HFpEF). Participants were stratified into tertiles based on TyG-BMI values. The primary composite endpoint included all-cause mortality and HF-related rehospitalization. Multivariable Cox proportional hazards models and restricted cubic spline analyses were employed to assess associations, with survival probabilities visualized using Kaplan–Meier estimates.

**Results:**

Over a median follow-up period of 52 months, 415 individuals died from all causes, and 479 experienced HF-related rehospitalization. TyG-BMI exhibited a U- or J-shaped association with long-term outcomes: both the lowest and highest TyG-BMI tertiles carried significantly higher risks of all-cause mortality and HF rehospitalization compared with the intermediate tertile, indicating that risk was not merely elevated at low values but followed a non-linear U- or J-shaped curve (*p* < 0.001). Restricted cubic spline analysis demonstrated an inverse J-shaped relationship between TyG-BMI and the composite endpoint, with inflection points at 193.10 for mortality and 222.30 for rehospitalization. Individuals in the lowest TyG-BMI tertile demonstrated a markedly elevated risk of the composite outcome (hazard ratio [HR]: 2.41, 95% confidence interval [CI]: 1.96–2.96, *p* < 0.001). These associations remained consistent in the HFpEF subgroup (*p* for interaction > 0.05). Additionally, TyG-BMI was strongly associated with all-cause mortality among individuals with concomitant right ventricular dysfunction (RVD) (HR: 2.34, 95% CI: 1.55–3.54, *p* < 0.001).

**Conclusion:**

A U-shaped association was observed: both very low and very high TyG-BMI were associated with increased all-cause mortality and HF rehospitalization. These findings support the potential utility of TyG-BMI as a prognostic biomarker for risk stratification. Furthermore, TyG-BMI appears to modify the relationship between RVD and adverse clinical outcomes, with obesity potentially exerting a modulatory effect.

## Introduction

Heart failure (HF) is a multifactorial clinical syndrome arising from a spectrum of underlying cardiovascular pathologies and is characterized by structural and functional myocardial impairments, as well as neurohormonal dysregulation (1, 2). It contributes substantially to global morbidity and mortality. In Western countries, HF accounts for approximately 1–2% of all hospital admissions, and its incidence is anticipated to increase in China due to demographic aging and a rising burden of chronic conditions such as coronary artery disease and hypertension (3, 4). Despite therapeutic advances, HF remains associated with high incidence rates, frequent rehospitalizations, and unfavorable prognoses, emphasizing the necessity for enhanced risk stratification tools and the identification of modifiable prognostic factors (5).

Insulin resistance (IR) has been recognized as a key pathophysiological mechanism in metabolic disorders, including diabetes mellitus, obesity, and metabolic syndrome, and has been closely associated with the onset and progression of HF (6, 7). A recent meta-analysis has confirmed the contributory role of IR in the development of HF (8). Although the hyperinsulinemic-euglycemic clamp remains the reference standard for quantifying IR, its clinical applicability is limited by technical complexity and resource requirements. Alternatively, the triglyceride glucose (TyG) index calculated from fasting triglyceride and glucose concentrations serves as a cost-effective and accessible surrogate marker of IR. Elevated TyG index levels have been significantly associated with increased risks of all-cause mortality and HF-related rehospitalization, indicating its potential utility as a prognostic indicator in individuals diagnosed with HF (9–11).

Recent evidence underscores the relevance of the TyG-body mass index (TyG-BMI), an integrated marker of metabolic dysfunction, in predicting cardiovascular risk (12–16). For instance, a population-based study conducted in Japan identified a positive association between TyG-BMI and the prevalence of hypertension (17). Analyses derived from the MIMIC-IV database have demonstrated an inverse relationship between TyG-BMI and mortality among critically ill individuals diagnosed with atrial fibrillation (18). Additionally, elevated TyG-BMI levels have been linked to an increased incidence of major adverse cardiovascular and cerebrovascular events following drug-eluting stent implantation, particularly among older adults and female individuals (19).

Right ventricular dysfunction (RVD) has emerged as a significant determinant of adverse outcomes in individuals with left-sided HF, adversely affecting prognosis in both HF with preserved ejection fraction (HFpEF) and HF with reduced ejection fraction (HFrEF) phenotypes (20). Systemic metabolic abnormalities, including obesity and IR, are increasingly recognized as modifiable contributors to RVD, potentially mediated through mechanisms such as myocardial fibrosis, inflammation, and lipotoxicity (21).

TyG-BMI integrates both dimensions: higher TyG reflects hepatic very-low-density lipoprotein over-production and impaired glucose uptake, whereas elevated BMI indicates systemic adipose-driven inflammation and sympathetic activation. Their product therefore quantifies the biological synergy between lipid toxicity and adipose-secretome stress, a combination known to accelerate myocardial fibrosis and right-ventricular after-load. Consequently, TyG-BMI shows a wider dynamic range and stronger dose–response gradients with hard outcomes than either TyG or BMI alone, making it a more sensitive single-number surrogate of IR-related HF risk.

In individuals with HFpEF and concomitant obesity, elevated pulmonary arterial pressures and right ventricular chamber dilation have been observed, contributing to biventricular dysfunction (22). Notably, a clinical study involving 15 women with obesity but no overt cardiovascular disease demonstrated that sustained weight reduction was associated with improved right ventricular function, underscoring the deleterious effects of metabolic dysfunction, particularly in individuals with elevated TyG-BMI on RVD (23).

Although several studies have examined the association between TyG-BMI and cardiovascular outcomes across various populations, its long-term prognostic relevance in individuals with HF remains insufficiently characterized. To address this gap, the present longitudinal cohort study was designed to evaluate the prognostic significance of TyG-BMI in a large sample of individuals with chronic HF. The study further aimed to assess its predictive performance across HF phenotypes and explore whether stratification by RVD enhances its prognostic performance.

The findings of this study may contribute to the development of targeted strategies for risk stratification and personalized management in HF and may offer novel insights into the clinical utility of TyG-BMI in predicting HF-related adverse outcomes.

## Methods

### Population and study design

We carried out this retrospective cohort study at Fuwai Central China Cardiovascular Hospital (a tertiary cardiovascular center in Central China) and enrolled every consecutive patient admitted to the Department of Cardiology with a diagnosis of chronic heart failure from December 2018 to December 2023. The inception cohort comprised consecutive patients who (i) were admitted to the Department of Cardiology, Fuwai Central China Cardiovascular Hospital, for the first time with a primary diagnosis of chronic heart failure (HF) between 1 December 2018 and 31 December 2023; (ii) had no previous HF-related hospitalizations in our institution; and (iii) met the 2018 Chinese HF guideline criteria. Patients entered the study on the date of this index admission (study baseline) and were followed up prospectively from that point onward. The diagnosis of HF was established in accordance with the 2018 clinical guidelines (24). Participants were stratified into three phenotypes based on left ventricular ejection fraction (LVEF): heart failure with preserved ejection fraction (HFpEF; LVEF ≥ 50%), heart failure with mid-range ejection fraction (HFmrEF; LVEF 40–49%), and heart failure with reduced ejection fraction (HFrEF; LVEF ≤ 40%).

Exclusion criteria were: (1) Age < 18 or >90 years; (2) history of acute coronary syndrome (ACS), percutaneous coronary intervention (PCI), or coronary artery bypass grafting (CABG) within the preceding month; (3) presence of congenital heart disease, moderate-to-severe valvular heart disease, acute myocarditis, idiopathic pulmonary hypertension (IPH), arrhythmogenic right ventricular cardiomyopathy (ARVC), hypertrophic cardiomyopathy, right ventricular myocardial infarction, pericardial disease, connective tissue disorders, thyroid dysfunction, or pregnancy; (4) estimated life expectancy < 1 year; (5) severe hepatic dysfunction or end-stage renal disease requiring chronic dialysis and/or estimated glomerular filtration rate (eGFR) < 15 mL/min/1.73 m^2^; and (6) missing essential clinical data.

**Figure 1:**
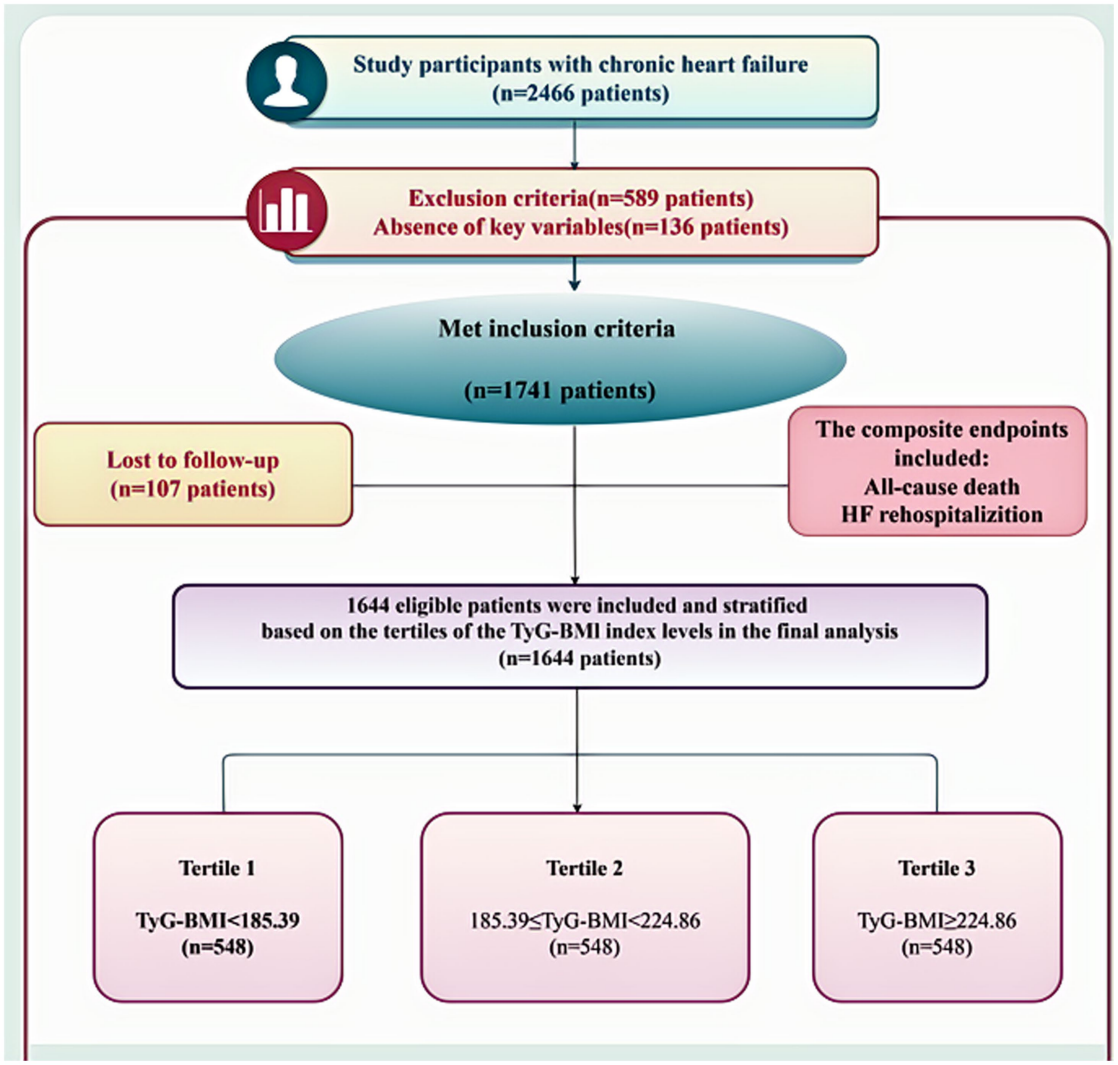
Representation of the protocol for participant selection in the research study. HF, heart failure; CHF, chronic heart failure; TyG-BMI, triglyceride-glucose-body mass index.

A flowchart depicting the enrollment process is illustrated in Figure 1. Following the exclusion of 136 individuals due to incomplete data and 107 due to loss to follow-up, 1,644 participants were included in the final analysis. Of these, 850 were classified as HFpEF, 223 as HFmrEF, and 571 as HFrEF. The attrition rate during follow-up was 6.1%. Participants were further stratified into tertiles based on TyG-BMI values: T1 (<185.39), T2 (185.39–224.85), and T3 (≥224.86).

All procedures conformed to the ethical standards of the institutional review board of Fuwai Central China Cardiovascular Hospital (Ethics Review No. 6 of 2022) and complied with the Declaration of Helsinki.

### Data collection and definitions

Clinical and demographic data were obtained from the institutional electronic medical records. Extracted variables included age, sex, BMI, history of smoking and alcohol use, vital signs, laboratory results, comorbid conditions, New York Heart Association (NYHA) functional class, scores from the Minnesota Living with Heart Failure Questionnaire (MLWHFQ), echocardiographic parameters, and prescribed pharmacologic therapies.

TyG-BMI was calculated as the product of the TyG index and BMI. The TyG index was computed as ln [fasting triglycerides (mg/dL) × fasting glucose (mg/dL)/2] (25). For unit conversions: 1 mmol/L of fasting glucose equals 18 mg/dL, and 1 mmol/L of triglycerides equals 88.6 mg/dL. BMI was calculated using the standard formula: body weight (kg) divided by height squared (m^2^).

Comorbidities such as chronic obstructive pulmonary disease (COPD), atrial fibrillation (AF), hypertension, diabetes mellitus (DM), dyslipidemia, and coronary heart disease (CHD) were defined using established clinical diagnostic criteria (26). The MLWHFQ was employed to assess HF–related quality-of-life domains, including social, physical, and emotional functioning. The albumin–bilirubin (ALBI) score was derived from serum albumin and total bilirubin levels, following validated formulas (26). Right ventricular dysfunction (RVD) was defined at baseline only using a prespecified echocardiographic algorithm adapted from the 2019 ASE/ESC guidelines: RVD present if any of the following were recorded within 24 h of admission: (1) TAPSE < 17 mm (primary criterion), (2) RV fractional area change (RVFAC) < 35% (secondary criterion, available in 92% of scans), (3) Systolic pulmonary arterial pressure (sPAP) > 35 mmHg plus RV dilatation (RV basal diameter > 41 mm) when tricuspid regurgitant envelope was measurable.

### Follow-up and endpoints

Participants were followed for a median of 52 months (interquartile range: 16–60 months). The primary composite endpoint included all-cause mortality and hospital readmission due to HF. Follow-up data were collected through structured telephone interviews conducted by trained physicians, corroborated by review of institutional medical records.

### Statistical analysis

Continuous variables were evaluated for normality. Normally distributed data are presented as mean ± standard deviation (SD) and compared using Student’s *t*-tests or one-way analysis of variance (ANOVA). Non-normally distributed data are expressed as median (IQR) and assessed using the Wilcoxon rank-sum test. Categorical variables were summarized as frequencies and percentages, with group differences assessed using appropriate chi-squared or Fisher’s exact tests.

Kaplan–Meier survival curves were used to estimate cumulative event rates across TyG-BMI tertiles. Comparisons were performed using the log-rank test. Restricted cubic spline (RCS) models were used to explore the non-linear association between TyG-BMI and clinical outcomes, with the median TyG-BMI value as the reference. Wald tests were used to evaluate overall and non-linear associations, with adjustments performed using the “rms” package in R software (version 4.4.1).

Univariate and multivariate Cox proportional hazards models were constructed to examine associations between TyG-BMI levels, analyzed both as continuous and categorical variables, and the risk of adverse clinical outcomes. Covariates were selected based on clinical relevance or a univariate significance threshold of *p* < 0.05. Prior to inclusion in multivariable models, multicollinearity among covariates was assessed.

Three hierarchical models were constructed to assess the association between TyG-BMI and adverse outcomes: Model 1 was adjusted for demographics and initial clinical variables, including sex, age, blood pressure, alcohol consumption, and smoking status. Model 2 included all covariates from Model 1 and further adjusted for comorbid conditions: hypertension, DM, CHD, dyslipidemia, COPD, and AF. Model 3 incorporated additional adjustments for laboratory parameters: Total cholesterol (TC), low-density lipoprotein cholesterol (LDL-C), high-density lipoprotein cholesterol (HDL-C), uric acid, glycated hemoglobin (HbA1c), CRP, N-terminal pro-brain natriuretic peptide (NT-ProBNP), estimated glomerular filtration rate (eGFR), and Galactin-3, as well as prescribed medications, including *β*-blockers, mineralocorticoid receptor antagonists, angiotensin receptor–neprilysin inhibitors (ARNIs), sodium-glucose cotransporter 2 (SGLT-2) inhibitors, statins, antidiabetics, diuretics, and digoxin. Model 3 was also adjusted for NYHA functional class, presence of RVD, and LVEF.

Subgroup analyses were conducted according to age (≤60 vs. >60 years), sex, LVEF (<50% vs. ≥50%), and presence of RVD. Interaction effects were assessed using likelihood ratio tests, with results visualized using forest plots. All statistical analyses were conducted using R software version 4.4.1 (The R Foundation, for Statistical Computing, Vienna, Austria). Two-sided *p*-values < 0.05 were considered statistically significant.

## Results

### Baseline characteristics

Baseline characteristics of the study population stratified by TyG-BMI tertiles are presented in Table 1 and Supplementary Table 1. The mean age of participants was 63 ± 11 years, with 56.2% identified as male. Individuals in the highest TyG-BMI tertile were younger and exhibited higher values for BMI, total cholesterol, LDL-C, HbA1c, cardiac troponin T, and Galectin-3 (all *p* < 0.001). The prevalence of DM, hypertension, and AF was comparable across tertiles. However, the use of SGLT_2_ inhibitors and digoxin was more frequent among participants in the lowest tertile.

**Table 1:** Study population characteristics according to TyG-BMI index tertiles.

Variables	All participants (*n* = 1,644)	Tertile 1 (*n* = 548)	Tertile 2 (*n* = 548)	Tertile 3 (*n* = 548)	*P*-value
Age, mean ± SD, (years)	63 ± 11	64 ± 12	64 ± 11	62 ± 12	0.536
Male, *n*(%)	924 (56.2%)	300 (54.7%)	320 (58.4%)	304 (55.5%)	0.436
BMI, mean ± SD, (Kg/m^2^)	23.88 ± 2.83	20.31 ± 1.58	23.48 ± 2.28	27.84 ± 2.79	<0.001
Hypertension, *n*(%)	780 (47.4%)	280 (51.1%)	253 (46.2%)	247 (45.1%)	0.104
Diabetes mellitus, *n*(%)	666 (40.5%)	219 (40.0%)	226 (41.2%)	221 (40.3%)	0.906
COPD, *n*(%)	225 (13.7%)	87 (15.9%)	73 (13.3%)	65 (11.9%)	0.147
Hyperlipidemia, *n*(%)	675 (41.1%)	188 (34.3%)	233 (42.5%)	254 (46.4%)	<0.001
Coronary heart disease, *n*(%)	721 (43.9%)	237 (43.2%)	226 (41.2%)	258 (47.1%)	0.141
Atrial fibrillation, *n*(%)	398 (24.2%)	131 (23.9%)	124 (22.6%)	143 (26.1%)	0.360
eGFR, median (IQR) (mL/min/1.73 m^2^)	67 (54, 82)	68 (55, 83)	69 (54, 82)	66 (54, 83)	0.426
Chlorine, mean ± SD, (mmol/L)	102 ± 5	101 ± 4	103 ± 3	105 ± 5	0.221
Cholesterol, mean ± SD, (mmol/L)	4.09 ± 1.16	3.85 ± 1.04	4.05 ± 1.08	4.35 ± 1.28	<0.001
LDL-c, mean ± SD, (mmol/L)	2.50 ± 0.84	2.35 ± 0.76	2.47 ± 0.84	2.68 ± 0.88	<0.001
NT-ProBNP, median(IQR) (pg/ml)	4,204 (3,024, 5,748)	4,240 (3,052, 5,759)	4,194 (3,021, 5,741)	4,164 (3,015, 5,744)	0.952
TG, median (IQR), (mmol/L)	1.18 (0.85, 1.79)	0.93 (0.73, 1.26)	1.25 (0.89, 1.86)	1.47 (1.09, 2.31)	<0.001
CRP, median (IQR), (mg/L)	7 (4, 12)	8 (4, 13)	7 (5, 12)	7 (4, 11)	0.760
E/e’, mean ± SD	19 ± 3	20 ± 4	18 ± 2	20 ± 3	<0.001
LVEF, *n*(%)					0.354
≤40%	571 (34.7%)	193 (35.2%)	192 (35.0%)	186 (33.9%)	
41–49%	223 (13.6%)	61 (11.1%)	81 (14.8%)	81 (14.8%)	
≥50%	850 (51.7%)	294 (53.6%)	275 (50.2%)	282 (51.3%)	
NYHA, *n*(%)					0.099
II	676 (41.1%)	217 (39.6%)	216 (39.4%)	243 (44.3%)	
III	892 (54.3%)	300 (54.7%)	314 (57.3%)	278 (50.7%)	
IV	76 (4.6%)	31 (5.7%)	18 (3.3%)	27 (4.9%)	
Glucose, median (IQR), (mmol/L)	5.3 (4.6, 6.6)	4.9 (4.4, 5.7)	5.4 (4.6, 6.9)	5.7 (4.8, 7.9)	<0.001
TyG-BMI, median (IQR)	207.01 ± 36.82	167.45 ± 13.05	203.85 ± 11.54	249.72 ± 19.09	<0.001

COPD, chronic obstructive pulmonary disease; eGFR, estimated glomerular filtration rate; LDL-C, low-density lipoprotein cholesterol; NT-ProBNP, N-terminal pro-B type natriuretic peptide; TG, triglyceride; CRP, C reactive protein; LVEF, left ventricular ejection fraction; BMI, body mass index; NYHA, New York Heart Association.

Although median levels of NT-proBNP levels were lower in the highest tertile, the difference was not statistically significant. The E/e’ ratio, a marker of diastolic dysfunction, was significantly elevated in the highest tertile (21 ± 3 vs. 20 ± 3, *p* < 0.001). Measures of right ventricular function, including tricuspid annular plane systolic excursion (TAPSE), pulmonary artery systolic pressure (PASP), and tricuspid regurgitant velocity (TRVmax), were significantly impaired in the highest TyG-BMI group (all *p* < 0.001).

### Composite endpoints and TyG-BMI

During the follow-up period, 415 participants (25.2%) experienced all-cause mortality, and 479 (29.1%) were rehospitalized due to HF. Cumulative mortality rates were 45.8% in T1, 17.8% in T2, and 36.4% in T3, while cumulative rehospitalization rates were 44.3, 24.8, and 30.9%, respectively.

**Figure 2:**
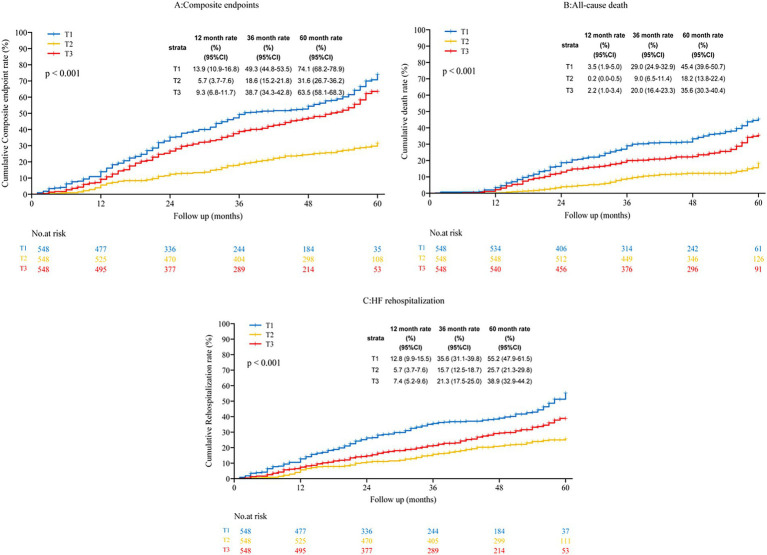
Kaplan–Meier estimation of various endpoints across tertiles of the TyG-BMI index. T1/T2/T3, first/s/third tertile of TyG-BMI; HF, heart failure; HR, hazard ratio; CI, confidence interval; **(A)** composite endpoints; **(B)** All-cause death; **(C)** Heart failure rehospitalization.

Kaplan–Meier survival analysis (Figure 2) demonstrated a significantly lower cumulative incidence of all-cause mortality and HF–related hospitalization among participants in the intermediate TyG-BMI tertile (Log-rank test, *p* < 0.001 for both endpoints).

**Figure 3:**
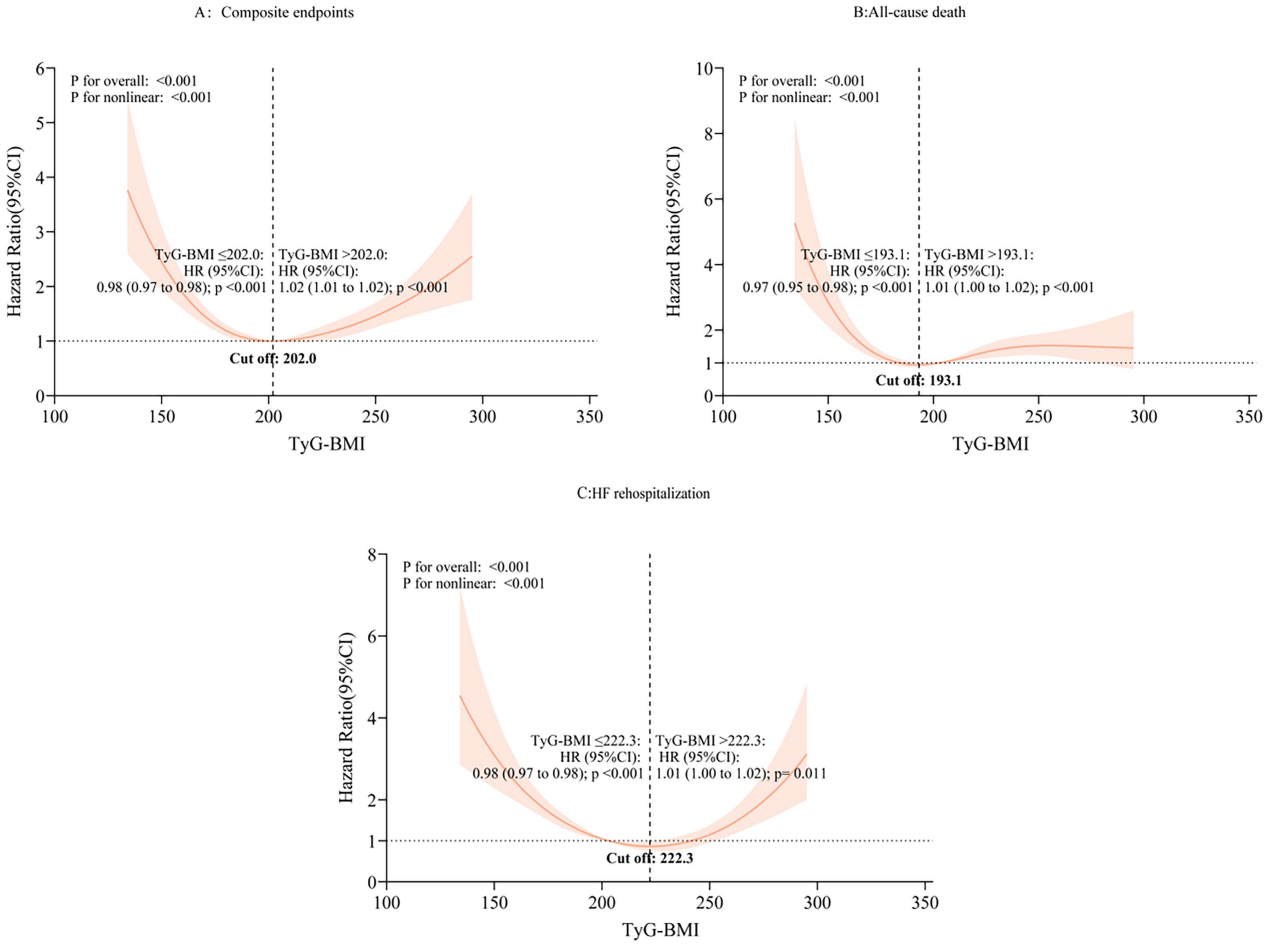
Non-linear correlations of the TyG-BMI index with various outcomes in the HF patients. RCS, restricted cubic spline; HR, hazard ratio; CI, confidence interval; TyG-BMI, triglyceride-glucose-body mass index; **(A)** composite endpoints; **(B)** All-cause death; **(C)** Heart failure rehospitalization.

The RCS analyses identified a non-linear association between TyG-BMI and adverse outcomes (*p* for non-linearity < 0.001) (Figure 3). All-cause mortality risk declined as the TyG-BMI value increased up to 193.10, after which risk rose (≤193.10: HR 0.97, 95% CI: 0.95–0.98; >193.10: HR 1.01, 95% CI: 1.00–1.02). Similarly, HF readmission risk decreased up to a TyG-BMI value of 222.30, then increased thereafter (≤222.30: HR 0.98, 95% CI: 0.97–0.98; >222.30: HR 1.01, 95% CI: 1.00–1.02).

**Figure 4:**
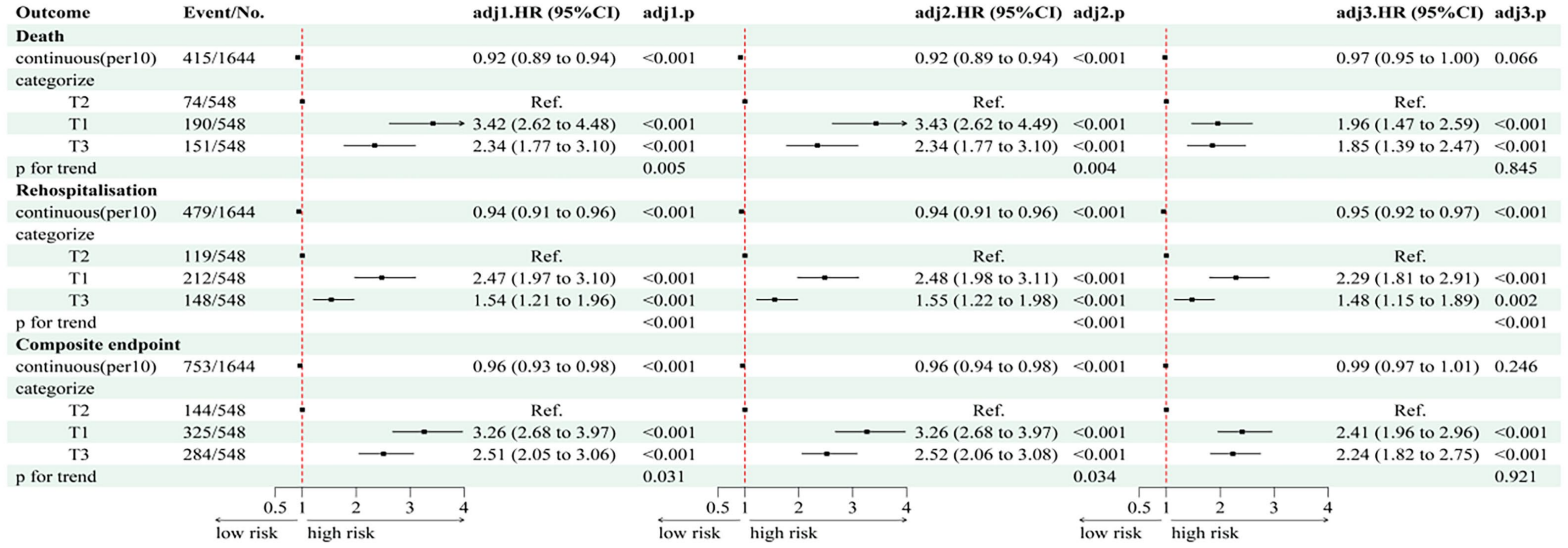
Multivariate cox results corresponding to the forest plot of TyG-BMI and composite endpoints. HR, hazard ratio; CI, confidence interval; RVD, right ventricular dysfunction; LVEF, left ventricular ejection fraction; T1/T2/T3, tertiles of TyG-BMI. Model 1 adjusted for gender, age, systolic and diastolic blood pressure, alcohol consumption and smoking practices. Model 2 adjusted for model 1 adds hypertension, DM, CHD, dyslipidemia, COPD; AF, trial fibrillation; DM, diabetes mellitus; CHD, coronary heart disease; COPD, chronic obstructive pulmonary disease; Model 3 adjusted for model 2 adds LDL-C, TC, HDL-C, HbA1c, UA, CRP, NT-ProBNP, eGFR, Gal-3, ARNI, SGLT-2i, β-blockers, MRA, antidiabetic drugs, statins, digoxin, diuretics, NYHA, RVD, LVEF. LDL-C, low-density lipoprotein cholesterol; HDL-C, high-density lipoprotein cholesterol; TC, total cholesterol; CRP, C-reactive protein; NT-proBNP, N-terminal pro-B-type natriuretic peptide; eGFR, estimated glomerular filtration rate; Gal-3, Galectin-3; UA, uric acid; NYHA, New York Heart Association; SGLT-2i, sodium–glucose cotransporter-2 inhibitor; MRA, mineralocorticoid receptor antagonist; ARNI, angiotensin receptor–neprilysin inhibitor.

Multivariate Cox regression analyses (Figure 4) demonstrated significantly higher risks of all-cause mortality in T1 and T3 compared to T2 (T1: HR 1.96, 95% CI: 1.47–2.59; T3: HR 1.85, 95% CI: 1.39–2.47; *p* < 0.001). Similar associations were observed for the composite endpoint of death and readmission (T1: HR 2.41, 95% CI: 1.96–2.96; T3: HR 2.24, 95% CI: 1.82–2.75; *p* < 0.001).

### Stratified analyses

**Figure 5:**
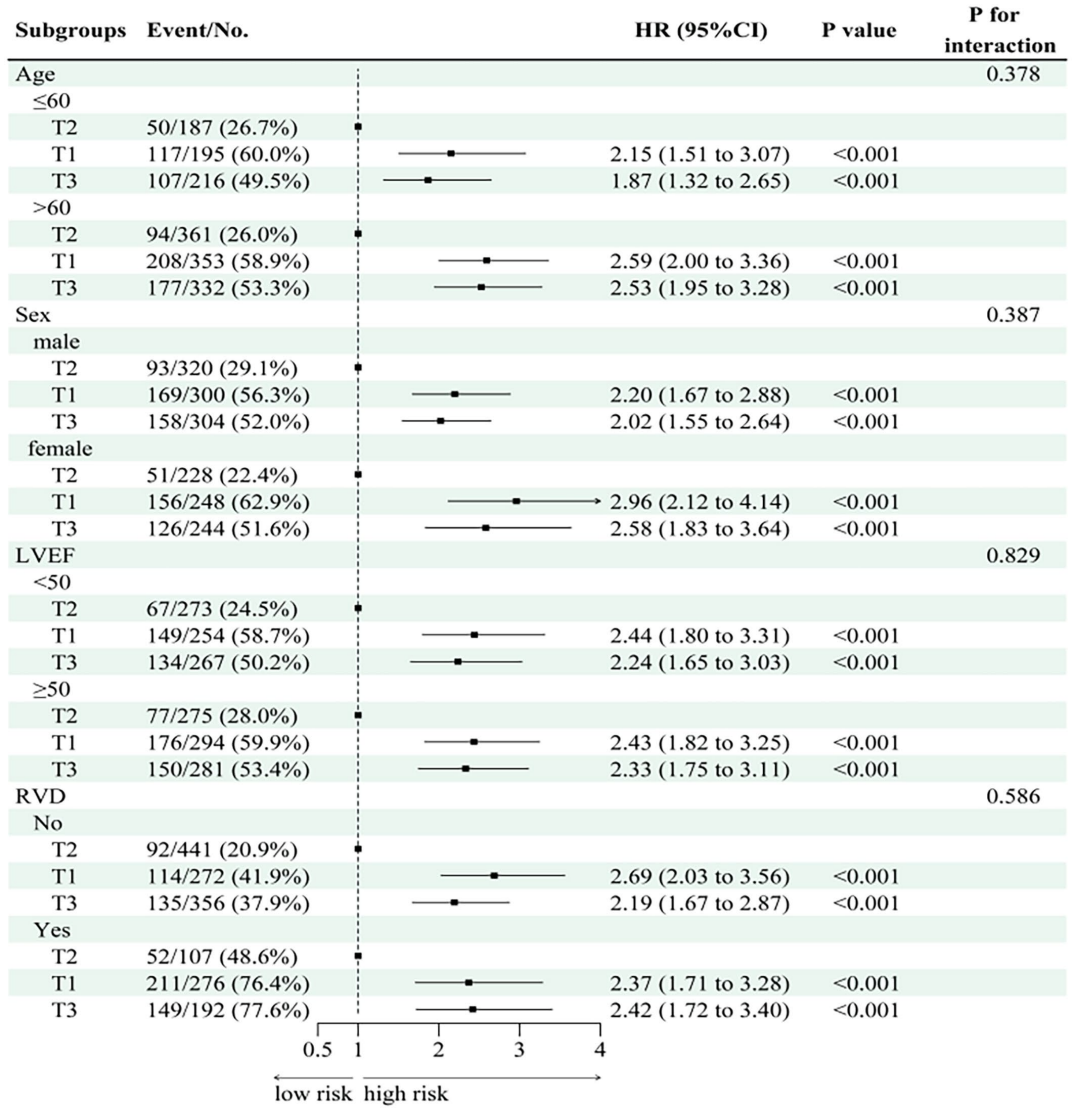
Subgroup analysis of TyG-BMI with respect to composite endpoints. HR, hazard ratio; CI, confidence interval; RVD, right ventricular dysfunction; LVEF, left ventricular ejection fraction; T1/T2/T3, tertiles of TyG-BMI.

**Figure 6:**
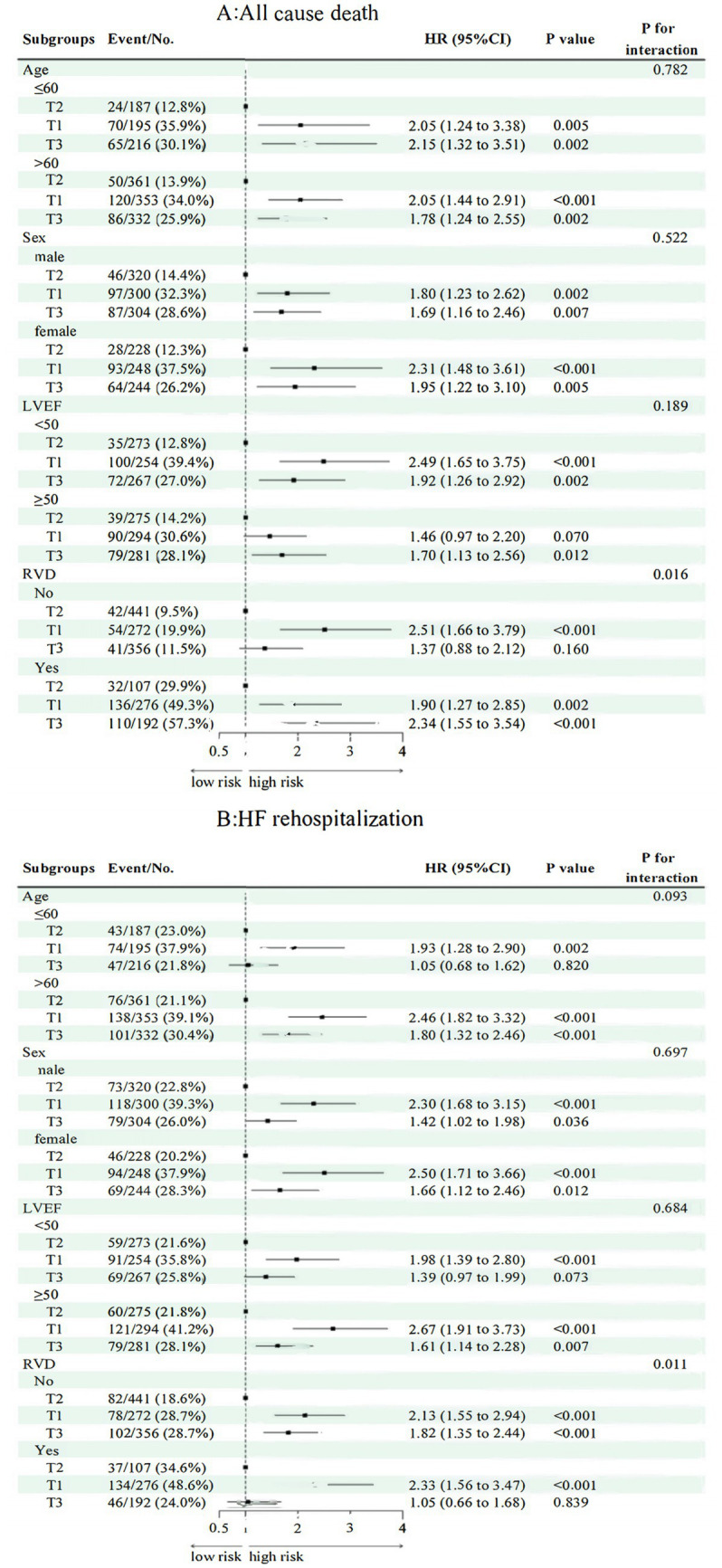
Subgroup analysis of TyG-BMI with respect to death from all causes and rehospitalization for HF. HR, hazard ratio; CI, confidence interval; RVD, right ventricular dysfunction; LVEF, left ventricular ejection fraction; T1/T2/T3, tertiles of TyG-BMI; **(A)** All cause death; **(B)** Heart failure rehospitalization.

Stratified analyses (Figures 5, 6) indicated consistent associations between TyG-BMI and adverse outcomes across subgroups defined by age, sex, and LVEF with no significant interactions detected (*p* for interaction = 0.829). Among participants with evidence of RVD, higher TyG-BMI values were significantly associated with increased all-cause mortality (HR: 2.34, 95% CI: 1.55–3.54, *p* < 0.001).

**Figure 7:**
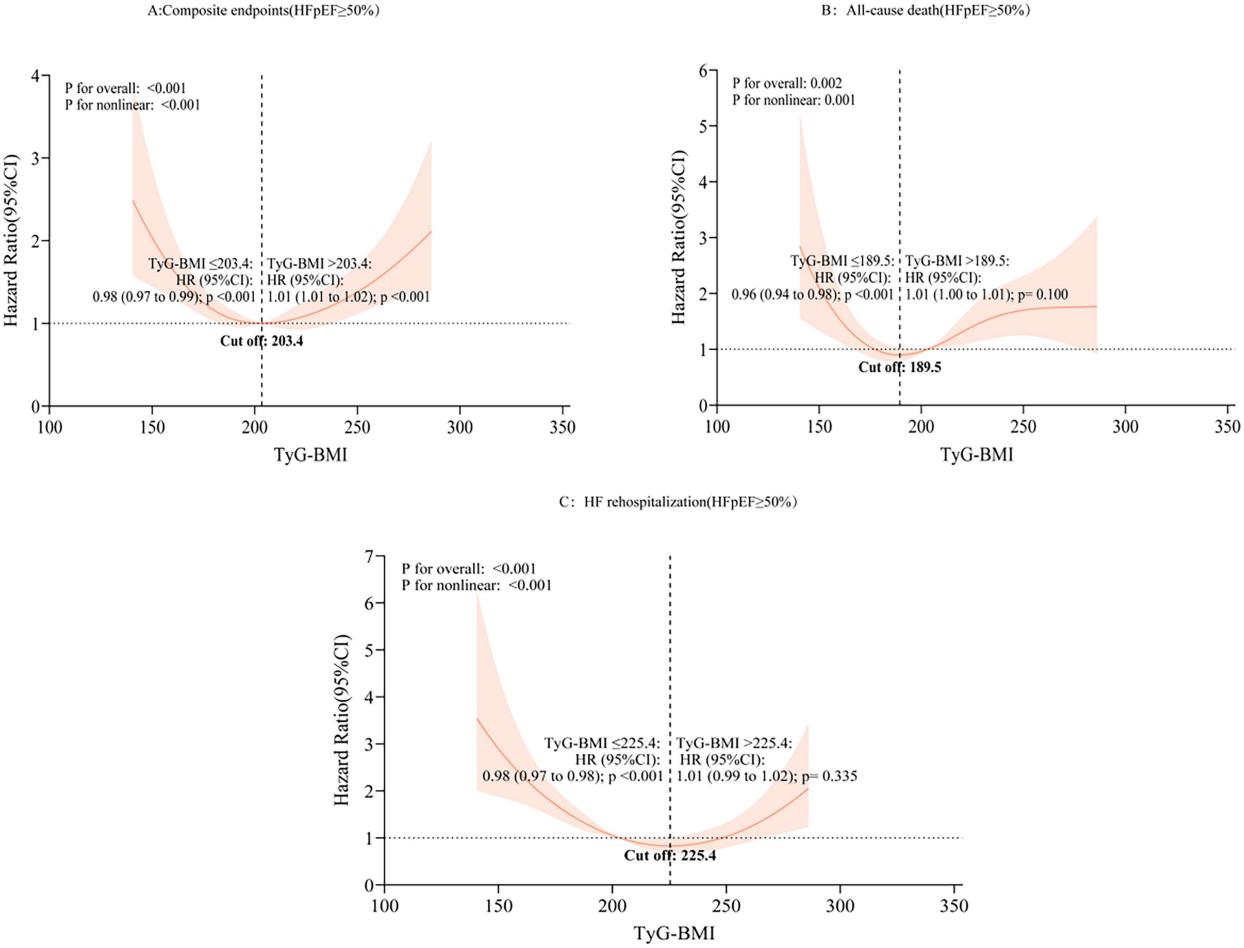
Non-linear association of TyG-BMI index with different prognoses in patients with HFpEF. HFpEF, heart failure with preserved ejection fraction; HR, hazard ratio; CI, confidence interval; TyG-BMI, triglyceride-glucose-body mass index; RCS, restricted cubic spline; **(A)** composite endpoints; **(B)** All-cause death; **(C)** Heart failure rehospitalization.

In RCS analysis conducted within the HFpEF subgroup (Figure 7), a non-linear relationship was observed between TyG-BMI and outcomes. Risk for the composite endpoint declined until a TyG-BMI value of 203.40, beyond which risk increased (≤203.40: HR 0.98, 95% CI: 0.97–0.99; >203.40: HR 1.01, 95% CI: 1.01–1.02). Similar inflection points were identified for all-cause mortality (cut-off: 189.50; HR 0.96, 95% CI: 0.94–0.98) and HF rehospitalization (cut-off: 225.40; HR 0.98, 95% CI: 0.97–0.98).

**Figure 8:**
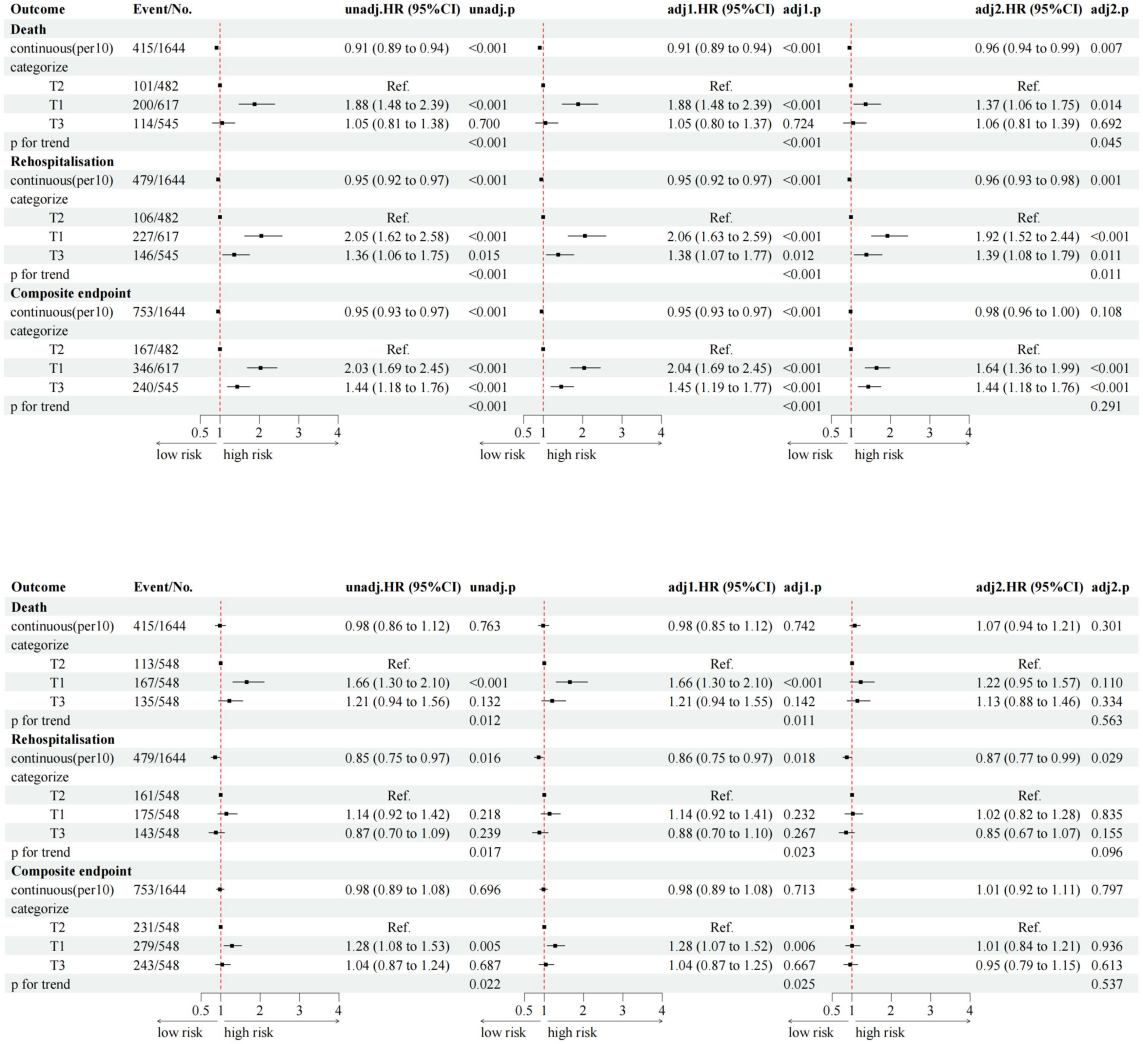
Multivariable Cox regression analyses of TyG index and BMI with clinical outcomes in heart failure patients.

**Figure 9:**
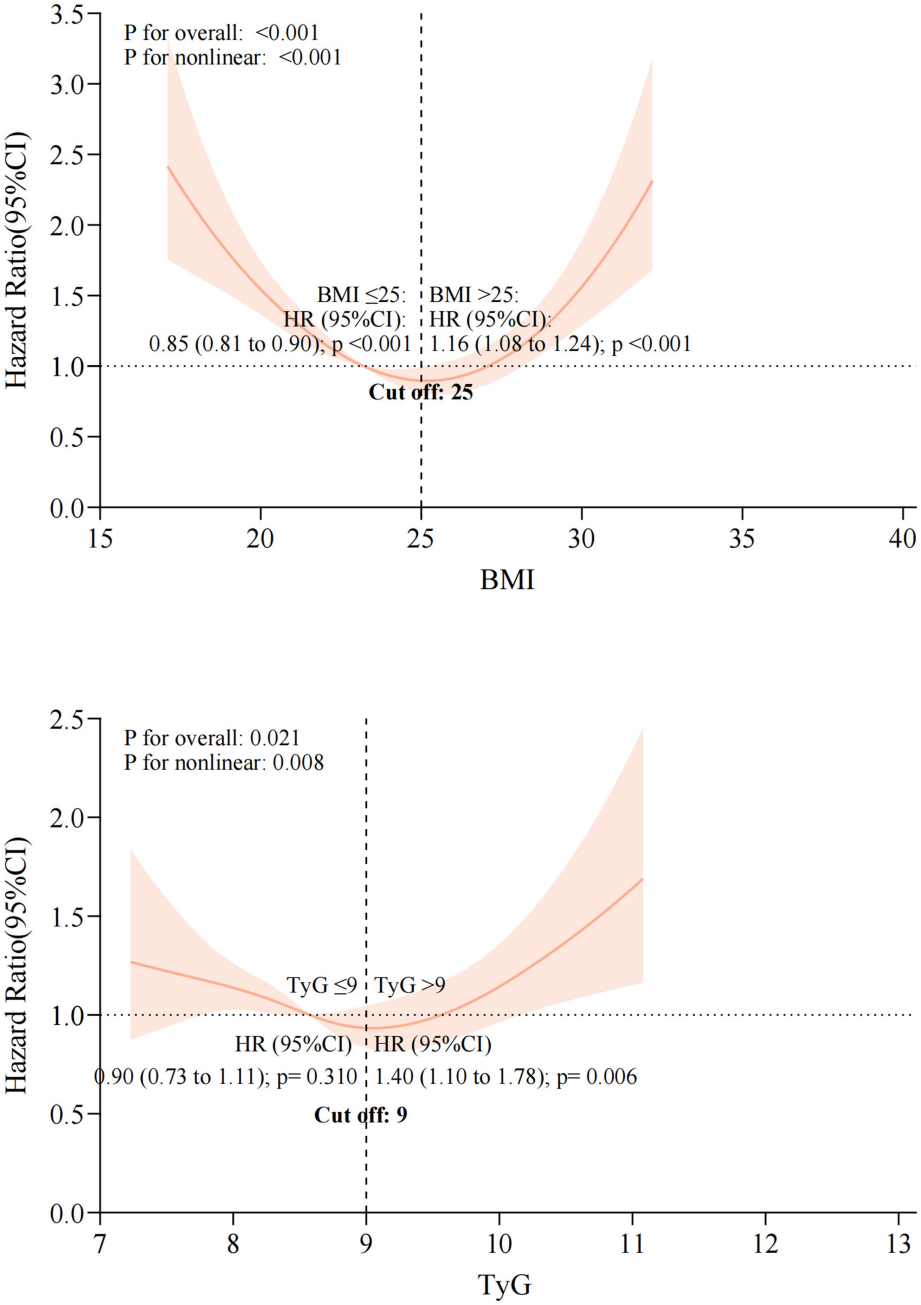
Restricted cubic spline analyses of the association between single-component indices and the risk of the composite endpoint.

The single-index models showed weaker predictive power than the combined TyG-BMI index, with lower hazard ratios and reduced statistical significance (Figure 8). This relationship was further illustrated by the markedly stronger non-linear associations of the TyG-BMI index with all clinical endpoints (Figure 9). Collectively, these findings support the superiority of the integrated TyG-BMI metric for risk stratification in patients with heart failure.

## Discussion

In this study, we identified a non-linear association between TyG-BMI and adverse clinical outcomes, including all-cause mortality and HF-related rehospitalization, in individuals with HF and varying levels of LVEF. Elevated TyG-BMI values were inversely associated with the incidence of composite adverse events after multivariable adjustment. Specifically, a reverse “J”-shaped association was observed with all-cause mortality, whereas a “U”-shaped association characterized the relationship with HF-related rehospitalization.

IR has been extensively implicated in the pathogenesis, progression, and adverse prognosis of HF (27). IR reduces tissue sensitivity to insulin, thereby impairing glucose uptake and disrupting metabolic homeostasis. In this setting, myocardial energy metabolism shifts from glucose to fatty acid oxidation. However, as IR worsens, myocardial capacity for fatty acid oxidation diminishes, resulting in lipid accumulation, increased oxidative stress, and cardiometabolic dysfunction. Additionally, IR amplifies the vasoconstrictive effects of angiotensin II, promoting cardiomyocyte hypertrophy and extracellular matrix deposition, which contribute to pathological cardiac remodeling and dysfunction (28, 29). IR further accelerates HF progression through the activation of the sympathetic nervous system and the rennin-angiotensin-aldosterone system, perpetuating myocardial fibrosis and adverse structural changes. Notably, HF can also exacerbate IR, creating a bidirectional pathophysiological cycle.

The prevalence of type 2 diabetes mellitus (T2DM) among individuals with HF ranges from 10 to 47%, and the incidence of newly diagnosed T2DM is significantly higher in the HF population compared to those without HF (30). Both systemic and myocardial IR have been reported among persons with HF, emphasizing the reciprocal relationship between metabolic dysregulation and HF progression (31).

The inverse relationship observed between TyG-BMI and all-cause mortality in this study may reflect the combined effects of IR and elevated BMI (32). IR is a well-established contributor to the development and progression of HF, and TyG-BMI has emerged as a validated surrogate marker for IR, with growing empirical support for its diagnostic utility (33, 34).

Approximately 70% of individuals with obesity demonstrate some degree of IR, and those with elevated BMI may possess a greater physiological reserve to counterbalance IR-related stressors compared to individuals with normal or low BMI (35). The phenomenon in which obesity appears to confer a protective effect in individuals with established HF, despite being a risk factor for its development, is commonly referred to as the “obesity paradox” (OP) (36). This paradox may account for the observed inverse association between TyG-BMI and all-cause mortality in the HF cohort. While elevated BMI is associated with increased HF incidence, its relationship with outcomes in those already diagnosed with HF is complex and often non-linear (37).

The prognostic significance of BMI in individuals with HF has been well documented (38). A large cohort study involving 108,927 individuals hospitalized for decompensated HF reported that each 5-unit increase in BMI was associated with a 10% reduction in in-hospital mortality (*p* < 0.001) (39). Similarly, a meta-analysis involving 22,807 participants demonstrated that individuals with low BMI exhibited the highest risk for both all-cause mortality and HF-related rehospitalization, whereas those classified as overweight exhibited the lowest risk (40).

Low TyG-BMI values may also reflect non-metabolic pathologies. A TyG-BMI in the bottom tertile frequently coincides with cardiac cachexia, systemic inflammation, protein-energy wasting and sarcopenia-conditions that are themselves powerful predictors of mortality. Because we did not measure grip strength, calf circumference, serum albumin, pre-albumin or other nutritional biomarkers, we cannot exclude the possibility that the elevated risk observed in the lowest TyG-BMI stratum is driven chiefly by reverse causation rather than by insulin resistance per se. Future studies should incorporate body-composition imaging, muscle-mass indices and dietary quality scores to disentangle the metabolic component of TyG-BMI from the cachexia/malnutrition component.

Several physiological mechanisms have been proposed to explain the OP. Higher BMI may mitigate the adverse effects of malnutrition-related inflammation and provide metabolic reserves during periods of catabolic stress (41, 42). Adipose tissue releases soluble tumor necrosis factor-alpha (TNF-*α*) receptors, which can neutralize the cytokine’s deleterious effects on myocardial tissue (43). Elevated lipoprotein levels in individuals with obesity may also attenuate systemic inflammation by neutralizing circulating lipopolysaccharides (44). Lower circulating BNP levels, often observed in individuals with obesity, may reflect better hemodynamic profiles, which could facilitate improved renal perfusion and enhanced tolerance to HF pharmacotherapies, such as ARNIs, SGLT2 inhibitors, and β-adrenergic receptor blockers (45). Greater adiposity may also support better cardiopulmonary reserve and muscular strength, further contributing to favorable outcomes in this subgroup (42).

Despite its established clinical relevance, the RV remains relatively understudied in the context of HF, due in part to the complexity of assessing RVD. TAPSE, a widely used echocardiographic marker of RV function, has been independently associated with improved outcomes at both baseline and follow-up, irrespective of RVD status (46). Obesity, while recognized as a major risk factor for HF and cardiovascular disease, has also been associated with impaired RV function. A study involving right heart catheterization demonstrated a consistent association between elevated BMI and RVD, with higher BMI levels correlating with increased risks of mortality and HF-related hospitalization (47).

Insulin resistance (IR) is a key driver of heart failure (HF) progression, yet its gold-standard measurement (hyper-insulinaemic clamp) is impractical in routine care. The triglyceride-glucose index (TyG) is a validated surrogate, but it ignores the anthropogenic component of IR. Multiplying TyG by BMI gives the TyG-BMI index, a single number that captures both lipid-toxic and adipose-secretory stress. In a 2025 population-based study of 8,753 U. S. adults with metabolic dysfunction-associated steatotic liver disease (MASLD), the TyG-BMI index demonstrated superior predictive performance for cardiovascular mortality compared with HOMA-IR, with the highest quartile of TyG-BMI showing an adjusted HR of 5.32 (95% CI: 2.12–7.84, *p* < 0.001) and AUC of 0.902, while HOMA-IR did not achieve statistical significance in the fully adjusted model (48).

Subgroup analyses from the present study are consistent with these findings, suggesting that BMI may modulate the influence the prognostic effect of RVD. Specifically, RVD appeared to exert a greater risk of mortality among individuals with higher BMI. These findings underscore the intricate interactions among obesity, RV function, and clinical outcomes in HF.

The findings of this study support the potential utility of TyG-BMI as a prognostic biomarker in individuals with HF. TyG-BMI showed a U−/J-shaped association with all-cause mortality, with both the lowest and highest tertiles carrying a higher risk than the intermediate group, underscoring the importance of routine monitoring of BMI and nutritional status across the entire TyG-BMI spectrum in this population. Loss of appetite and unintentional weight loss, often associated with gastrointestinal edema, may precede overt HF decompensation. Thus, early nutritional assessment and intervention, combined with individualized risk stratification incorporating TyG-BMI, may enhance clinical outcomes. These findings highlight the complex interplay between metabolic derangements and HF progression.

### Study limitations

Several limitations should be acknowledged when interpreting the findings of this study. First, the analysis was based on baseline data collected at the time of hospital admission; longitudinal measurements of metabolic or biomarker parameters were not available. Second, the lack of direct comparative data limited the ability to evaluate the TyG-BMI against other established surrogate markers of IR. Third, although consecutive enrolment and completeness of follow-up were high, all patients were recruited from a single center; thus, referral bias, regional lifestyle factors, and center-specific protocols could limit external validity, and the findings may not be generalisable to populations with different ethnic compositions, dietary patterns, or health-care infrastructures. In addition, although the sample size provided adequate statistical power, the relatively short follow-up duration may introduce further selection bias. Multi-center validation is therefore mandatory before TyG-BMI can be adopted as a routine risk-stratification tool, and future prospective, multicenter studies with extended follow-up are warranted to validate these results.

## Conclusion

The TyG-BMI index may serve as a simple, clinically useful prognostic marker for risk stratification in HF patients. However, given the single-center design, multicentre validation is essential before clinical implementation. This study identified a significant non-linear association between TyG-BMI and adverse clinical outcomes, specifically all-cause mortality and HF-related rehospitalization, among individuals with HF, including those across the full spectrum of LVEF phenotypes. These associations persisted over long-term follow-up and consistent with previously reported findings. TyG-BMI may serve as a clinically relevant biomarker for risk stratification and therapeutic decision-making in HF management. However, its routine clinical application requires further validation through large-scale prospective studies aimed at evaluating its predictive accuracy and integration into established risk stratification algorithms.

## Data Availability

The raw data supporting the conclusions of this article will be made available by the authors, without undue reservation.

## Glossary

HF: heart failure
CHF: chronic heart failure
HFpEF: heart failure with preserved ejection fraction
HFrEF: heart failure with reduced ejection fraction
HFmrEF: heart failure with mildly reduced ejection fraction
TyG-BMI: triglyceride-glucose-body mass index
RVD: right ventricular dysfunction
LVEF: left ventricular ejection fraction
TAPSE: tricuspid annular plane systolic excursion
sPAP: systolic pulmonary artery pressure
NT-proBNP: N-terminal pro-B-type natriuretic peptide
BMI: body mass index
HR: hazard ratio
CI: confidence interval
NYHA: New York Heart Association
eGFR: estimated glomerular filtration rate
COPD: chronic obstructive pulmonary disease
AF: trial fibrillation
DM: diabetes mellitus
CHD: coronary heart disease
TC: Total cholesterol
LDL-C: low-density lipoprotein cholesterol
HDL-C: high-density lipoprotein cholesterol
CRP: C-reactive protein
HbA1c: glycated hemoglobin
SGLT-2i: sodium–glucose cotransporter-2 inhibitor
MRA: mineralocorticoid receptor antagonist
ARNI: angiotensin receptor–neprilysin inhibitor
