# Supplementary material for: Association between triglyceride glucose-body mass index and long-term adverse outcomes in individuals with heart failure: a retrospective cohort study
Source: Front Nutr. 2025 Nov 24;12:1688566. doi: 10.3389/fnut.2025.1688566 (PMC12682654; doi:10.3389/fnut.2025.1688566)
Supplement: Supplementary file 1 [file Table_1.docx]

**Supplementary material**

1. **Supplementary table 1 Study population characteristics according to TyG-BMI index tertiles**

| **Variables** | **All participants**  **(n=1644)** | **Quartile 1 (n=548)** | **Quartile 2 (n=548)** | **Quartile 3 (n=548)** | **P value** |
| --- | --- | --- | --- | --- | --- |
| Age,mean±SD, (years) | 63 ± 11 | 64 ± 12 | 64 ± 11 | 62 ± 12 | **0.536** |
| Male,n(%) | 924 (56.2%) | 300 (54.7%) | 320 (58.4%) | 304(55.5%) | **0.436** |
| Smoke,n(%) | 426(25.9%) | 142(25.9%) | 136(24.8%) | 148(27.0%) | **0.710** |
| Drinking,n(%) | 240(14.6%) | 81(14.8%) | 70(12.8%) | 89(16.2%) | **0.264** |
| Hypertension,n(%) | 780 (47.4%) | 280 (51.1%) | 253 (46.2%) | 247 (45.1%) | **0.104** |
| Diabetes mellitus,n(%) | 666 (40.5%) | 219 (40.0%) | 226 (41.2%) | 221 (40.3%) | **0.906** |
| COPD,n(%) | 225 (13.7%) | 87(15.9%) | 73(13.3%) | 65 (11.9%) | **0.147** |
| Hyperlipidemia,n(%) | 675 (41.1%) | 188 (34.3%) | 233 (42.5%) | 254 (46.4%) | **< 0.001** |
| Coronary heart disease ,n(%) | 721 (43.9%) | 237 (43.2%) | 226 (41.2%) | 258 (47.1%) | **0.141** |
| Atrial fibrillation,n(%) | 398 (24.2%) | 131(23.9%) | 124 (22.6%) | 143(26.1%) | **0.360** |
| Systolic blood pressure  mean±SD, (mmHg) | 120±18 | 121±19 | 122±20 | 119±17 | **0.897** |
| Diastolic blood pressure  mean±SD,(mmHg) | 79±12 | 78±12 | 79±11 | 80±13 | **0.720** |
| Heart rate,mean±SD,(bpm) | 83±13 | 82±12 | 81±10 | 83±11 | **0.452** |
| Hemoglobin,mean±SD,(g/L) | 127±19 | 126±17 | 128±16 | 129±15 | **0.456** |
| eGFR,median(IQR)  (mL/min/1.73 m^2^) | 67(54,82) | 68(55,83) | 69(54,82) | 66(54,83) | **0.426** |
| Sodium,mean±SD,(mmol/L) | 139±5 | 138±4 | 140±3 | 137±6 | **0.276** |
| Chlorine,mean±SD,(mmol/L) | 102±5 | 101±4 | 103±3 | 105±5 | **0.221** |
| Cholesterol,mea±SD,(mmol/L) | 4.09±1.16 | 3.85±1.04 | 4.05±1.08 | 4.35±1.28 | **< 0.001** |
| LDL-c,mean±SD,(mmol/L) | 2.50±0.84 | 2.35±0.76 | 2.47±0.84 | 2.68±0.88 | **< 0.001** |
| ST_2_,median (IQR),(ng/ml) | 31(24,41) | 33(25,42) | 30(23,40) | 32(25,42) | **0.394** |
| Gal-3,median (IQR),(ng/ml) | 15(13,21) | 16(13,21) | 15(12,20) | 17(14,22) | **< 0.001** |
| FT3,median (IQR),(pmol/L) | 4.26(3.74,4.83) | 4.35(3.77,4.88) | 4.23(3.66,4.81) | 4.24(3.75,4.29) | **0.313** |
| FT4,median (IQR),(pmol/L) | 16.74(14.92,18.86) | 16.75(14.91,19.10) | 16.74(14.90,18.53) | 16.72(14.94,19.08) | **0.603** |
| TSH,median (IQR),(mIU/L) | 2.17(1.29,3.20) | 2.03(1.19,3.12) | 2.25(1.33,3.35) | 2.19(1.33,3.22) | **0.575** |
| cTNT,median (IQR),(pg/ml) | 31.33(25.21,40.42) | 35.32(24.56,51.15) | 27.03(24.39,31.13) | 36.34(27.53,39.42) | **< 0.001** |
| NT-ProBNP,median(IQR)  (pg/ml) | 4204(3024,5748) | 4240(3052,5759) | 4194(3021,5741) | 4164(3015,5744) | **0.952** |
| TG,median (IQR),(mmol/L) | 1.18(0.85,1.79) | 0.93 (0.73, 1.26) | 1.25 (0.89, 1.86) | 1.47 (1.09, 2.31) | **< 0.001** |

| **Variables** | **All participants**  **(n=1644)** | **Quartile 1 (n=548)** | **Quartile 2 (n=548)** | **Quartile 3 (n=548)** | **P value** |
| --- | --- | --- | --- | --- | --- |
| Uric acid,median(IQR) (mmol/L) | 425(389,473) | 427(387,476) | 424(386,472) | 421(396,472) | **0.729** |
| CRP,median (IQR),(mg/L) | 7(4,12) | 8(4,13) | 7(5,12) | 7(4,11) | **0.760** |
| Glucose,median(IQR),(mmol/L) | 5.3(4.6,6.6) | 4.9(4.4,5.7) | 5.4(4.6,6.9) | 5.7(4.8,7.9) | **< 0.001** |
| ALBI score,mean±SD | -2.40±0.46 | -2.39±0.37 | -2.41±0.35 | -2.42±0.36 | **0.224** |
| LVEDD,mean±SD,(mm) | 63±7 | 62±8 | 61±6 | 64±9 | **0.053** |
| LAVI,mean±SD,(mL/m^2^) | 38±4 | 37±5 | 36±6 | 39±7 | **0.960** |
| CI,mean±SD,(L/min/m^2^) | 2.57±0.44 | 2.54±0.45 | 2.56±0.47 | 2.59±0.48 | **0.221** |
| TAPSE,mean±SD,(mm) | 18±2 | 17±1 | 19±3 | 16±3 | **< 0.001** |
| PASP,mean±SD,(mmHg) | 45±8 | 46±6 | 44±7 | 48±8 | **< 0.001** |
| RV S’,mean±SD,(cm/s) | 8.7±2.1 | 8.6±2.0 | 8.9±2.2 | 8.8±1.9 | **0.059** |
| RVFAC ,mean±SD,(%) | 34.67±6.01 | 34.94±5.78 | 34.85±5.84 | 34.22±6.36 | **0.100** |
| TRV max ,mean±SD,(cm^2^) | 4.97±1.99 | 4.92±1.09 | 4.72±1.08 | 5.29±1.55 | **< 0.001** |
| E/e’,mean±SD | 19±3 | 20±4 | 18±2 | 20±3 | **< 0.001** |
| LVEF,n(%) |  |  |  |  | **0.354** |
| ≤40% | 571(34.7%) | 193(35.2%) | 192(35.0%) | 186(33.9%) |  |
| 41-49% | 223(13.6%) | 61(11.1%) | 81(14.8%) | 81(14.8%) |  |
| ≥50% | 850(51.7%) | 294(53.6%) | 275(50.2%) | 282(51.3%) |  |
| HbA1C,median (IQR),(mmol/L) | 5.30 (4.61, 6.58) | 4.94 (4.43, 5.67) | 5.43 (4.63, 6.87) | 5.70 (4.87, 7.99) | **< 0.001** |
| BMI,mean±SD,(Kg/m^2^) | 23.88 ± 2.83 | 20.31 ± 1.58 | 23.48 ± 2.28 | 27.84 ± 2.79 | **< 0.001** |
| TyG-BMI,median (IQR) | 207.01 ± 36.82 | 167.45± 13.05 | 203.85 ± 11.54 | 249.72± 19.09 | **< 0.001** |
| Statins,n(%) | 745 (45.3%) | 231 (42.2%) | 242 (44.2%) | 272 (49.6%) | **0.053** |
| Digoxin,n(%) | 636 (38.7%) | 248 (45.3%) | 192 (35.0%) | 196 (35.8%) | **< 0.001** |
| Diuretics,n(%) | 1343 (81.7%) | 460 (83.9%) | 437 (79.7%) | 446 (81.4%) | **0.194** |
| SGLT2i,n(%) | 729 (44.3%) | 211 (38.5%) | 238 (43.4%) | 280 (51.1%) | **< 0.001** |
| ARNI,n(%) | 1303 (79.3) | 420 (76.6) | 447 (81.6) | 436 (79.6) | **0.129** |
| β-blocks,n(%) | 1376 (83.7%) | 452 (82.5%) | 453 (82.7%) | 471 (85.9%) | **0.217** |
| MRA,n(%) | 1271 (77.3%) | 428 (78.1%) | 418 (76.3%) | 425 (77.6%) | **0.760** |
| NYHA,n(%) |  |  |  |  | **0.099** |
| Ⅱ | 676 (41.1%) | 217 (39.6%) | 216 (39.4%) | 243 (44.3%) |  |
| Ⅲ | 892 (54.3%) | 300 (54.7%) | 314 (57.3%) | 278 (50.7%) |  |
| Ⅳ | 76 (4.6%) | 31 (5.7%) | 18 (3.3%) | 27 (4.9%) |  |
| MLWHFQ,mean±SD | 51±4 | 53±2 | 50±3 | 52±3 | **0.455** |

Abbreviations: COPD,chronic obstructive pulmonary disease;eGFR, estimated glomerular filtration rate; LDL-C, low-density lipoprotein cholesterol; ST2,growth stimulation to express the gene 2;Gal-3,galectin-3;NT-ProBNP, N-terminal pro-B type natriuretic peptide;TG,triglyceride;CRP, C reactive protein;

ALBI,Albumin-bilirubin;LVEDD, left ventricularend-diastolic diameter; LAVI,left atrial volume index;CI,cardiac index;TAPSE,tricuspid annular plane systolic excursion;PASP,pulmonary artery systolic pressure;RVs’,right ventricular peak systolic S’ velocity;RVFAC,right ventricular fractional area change;TRV,tricuspid regurgitation; LVEF, left ventricular ejection fraction; BMI, body mass index;TyG, triglyceride glucose;SGLT2i, sodium-glucose transporter 2 inhibitor;ARNI, angiotensin receptor neprilysin inhibitor; MRA,mineralocorticoid receptor inhibitor;NYHA, New York Heart Association; MLWHFQ,minnesota Living with Heart Failure Questionnaire.
